# A New Evidential Reasoning Rule Considering Evidence Correlation with Maximum Information Coefficient and Application in Fault Diagnosis

**DOI:** 10.3390/s25103111

**Published:** 2025-05-14

**Authors:** Shanshan Liu, Guanyu Hu, Shaohua Du, Hongwei Gao, Liang Chang

**Affiliations:** 1 Guangxi Key Laboratory of Trusted Software, Guilin University of Electronic Technology, Guilin 541004, China; 22031202014@mails.guet.edu.cn (S.L.); 21032202007@mails.guet.edu.cn (S.D.); 22032303020@mails.guet.edu.cn (H.G.); 2 Key Laboratory of the Ministry of Education, Guilin University of Electronic Technology, Guilin 541004, China; huguanyu@guet.edu.cn

**Keywords:** evidential reasoning rule, correlation, maximum mutual information coefficient, fault diagnosis

## Abstract

The evidential reasoning (ER) rule has been widely adopted in engineering fault diagnosis, yet its conventional implementations inherently neglect evidence correlations due to the foundational independence assumption required for Bayesian inference. This limitation becomes particularly critical in practical scenarios where heterogeneous evidence collected from diverse sensor types exhibits significant correlations. Existing correlation processing methods fail to comprehensively address both linear and nonlinear correlations inherent in such heterogeneous evidence systems. To resolve these theoretical and practical constraints, this study develops MICER—a novel ER framework that incorporates correlation analysis based on the maximum mutual information coefficient (MIC). The proposed methodology advances ER theory by systematically integrating evidence interdependencies, thereby expanding both the theoretical boundaries of ER rules and their applicability in real-world fault diagnosis. Flange ring loosening fault diagnosis and flywheel system fault diagnosis cases are experimentally verified and the effectiveness of the method is demonstrated.

## 1. Introduction

Evidence theory has been significantly advanced in multi-source data integration in recent years [1,2]. As the cornerstone of the Dempster–Shafer (D-S) theory, the combination rule has been established as the fundamental mechanism in foundational evidence theory. Despite its robust evidence fusion capabilities, the emergence of counter-intuitive outcomes under conflicting evidence conditions has been widely documented [3,4]. The foundational breakthrough emerged with Yang and Singh’s [5] formulation of normalized evidence weighting, which was subsequently refined by Yang and Xu [6] through the ER Algorithm (ERA) specifically designed to resolve counter-intuitive outcomes in conflicting evidence scenarios.

Building upon the ERA framework, the ER rule [7] has been developed to extend both Dempster–Shafer evidential theory [8] and reasoning methodologies [9]. The ER rule operationalizes evidence synthesis through a generalized probabilistic reasoning framework. This dual-parameter integration systematically combines evidential weight with evidential reliability within a unified probabilistic framework. Evidential weight is determined by relative importance evaluation guided by decision-maker preferences to characterize subjective uncertainty. In parallel, evidential reliability is defined as the evidence source’s capacity for accurate measurement, serving as an objective uncertainty metric. Through orthogonal parameter treatment, reliability and weight are conjunctively embedded in belief distribution modeling. For fuzzy semantic inputs, affiliation functions are systematically applied for uncertainty-preserving information transformation. This synthesis establishes the ER rule as a comprehensive methodology for uncertainty quantification, fuzzy information processing, and probabilistic inference.

Since its introduction, the ER rule has been extensively studied due to its demonstrated scalability and adaptability. The power set domains ER (PSA_ER) framework was enhanced by Wang et al. [10] through attribute-based evidence integration, while the FoD framework was extended to PSA_ER. Mathematical formulations for dependability and evidence weight were established, along with physical interpretations of model parameters. Parameter optimization was subsequently performed using intelligent optimization techniques to enhance PSA_ER’s performance. A novel ER rule was developed by Du et al. [11] to address weight–reliability interdependencies, where evidence was recursively combined through orthogonal sum operations with formal theorems and inferences being established. Verification was conducted through numerical comparisons and case studies demonstrating the proposed methods’ effectiveness. ERr-CR was proposed by Wang et al. [12] for continuous reliability distributions, where reliability was defined as a random variable with probability distributions for paired evidence sources. Expected utility theory was introduced to characterize ERr-CR outputs. The framework was extended to multi-evidence systems, while an ER rule considering multi-source parameter uncertainties was formulated by Wang et al. [13]. A unified inference model was developed based on ER rule principles.

Meanwhile, ER has been extensively implemented in engineering domains including performance evaluation [14], pattern classification [15], and especially in the field of fault diagnosis. Based on ER rules, a new concurrent fault diagnosis model is proposed by Ning et al. [16]. Multiple sub-ER models are built, forming parallel diagnostic patterns, and thus system reliability is improved. An ER-based time–space domain cascade fusion model (TS-ER for short) is proposed by Xu et al. [17] for rudder fault diagnosis. It can obtain both time domain fusion evidence and local fusion evidence given by different spatial locations to obtain joint diagnostic evidence in both time and space domains. Based on the set-theoretic correlation measure, a regularization term is added by [18] to compute the relationship between the internal data so that rotating machinery fault diagnosis is realized. An unbalanced ensemble approach with DenseNet and evidential inference rules is proposed by Wang et al. [19] and is used for diagnosing mechanical faults in the case of class unbalance. A fault diagnosis model based on evidential reasoning (ER) rules is proposed in [20] for fault diagnosis of oil-immersed transformers. Its reference points are in the form of Gaussian distribution and optimized by a constrained genetic optimization algorithm (GA). For monitoring-based bearing fault diagnosis, a novel diagnostic ensemble approach with evidential inference rules was proposed by Wang et al. [21], which can reduce the negative impact of interrelated and redundant features as well as generate accurate and diverse base classifiers. An online monitoring and fault diagnosis method for capacitor aging based on evidential reasoning (ER) rules was introduced by Liao et al. [22]. It extracts features from DC link voltage data with different capacitor aging levels and generates data features as diagnostic evidence, which are then combined according to ER rules. Finally, the combination results are used to estimate the capacitor aging fault level. Atanasoff interval-valued intuitionistic fuzzy sets and the belief rule base are combined by Jia et al. [23] to develop an ER fault detection model for fault detection in flush airborne data sensing. A new multimodal recognition framework based on an evidential reasoning approach with interval reference values (ER-IRV) is constructed in [24] for multimodal system fault detection.

The effectiveness of ER rules has been demonstrated across diverse application scenarios through these empirical investigations. The Bayesian probabilistic foundation of ER rules requires evidence independence, which may be violated in complex systems with inherent indicator correlations. Novel interdependent reasoning frameworks have been developed, most notably the MAKER framework [25] designed for data-driven modeling under hybrid uncertainty conditions. Quantitative measurement was achieved for both evidence reliability and pairwise interdependencies. Engineering validation was conducted in [26], with the MAKER framework’s implementation process detailed in Section 2.1. A MAKER-enhanced classifier architecture is developed by He et al. [27] for feature relevance extraction and classification optimization. The ERr-DE framework is established in [28] for dependent evidence integration. Aggregation sequencing is optimized based on evidence reliability metrics. A distance correlation methodology was implemented to compute Relative Total Dependence Coefficients (RTDC). The RTDC mechanism was embedded as a discount factor within the ERr-DE framework’s probabilistic architecture. However, in the fault diagnosis of complex engineering systems, there is a temporal or spatial correlation between the collected indicators due to different sensor types. For example, because the sensor deployment distance is too close, the sensor perception will be crossed, and there will be a spatial correlation between the indicators used in fault diagnosis; when some fault diagnosis such as the influence of weather factors needs to be considered, there is a temporal correlation between the temperature and humidity indicators that are collected by the sensors at different times. Current computational methodologies fail to account for this heterogeneity, resulting in biased correlation estimates. This critical limitation is observed in [26], but nonlinear associations are not considered in the analysis of evidence relevance.

Despite significant advancements in evidential reasoning (ER) applications for fault diagnosis, critical limitations persist in addressing evidence correlations: **Independence Assumption Incompatibility**: State-of-the-art ER frameworks ([16,17,18,19,20,21,22,23,24]) inherently rely on the Bayesian independence axiom, disregarding intrinsic sensor network interactions (e.g., thermal–stress coupling in flange systems [16], spatiotemporal rudder fault couplings [17]). While the MAKER framework [25] partially addresses hybrid uncertainties, it retains this restrictive assumption, leading to systematic deviations in multi-sensor systems. **Parametric Constraints in Correlation Modeling:** Existing correlation-aware ER variants ([18,20]) impose distributional assumptions (Gaussian priors in [18], GA-optimized reference points in [20]), failing to capture nonlinear interdependencies among heterogeneous evidence (e.g., vibration spike distributions vs. chemical sensor Poisson noise). **Homogeneous Evidence Bias:** Current methodologies predominantly focus on single-modality fusion (e.g., voltage-based capacitor aging in [22]), neglecting metric space incompatibility between heterogeneous sensors (vibration/thermal/chemical) and quantification challenges for cross-modal evidence (interval-valued fuzzy sets [23] vs. temporal ER-IRV [24]). These gaps fundamentally restrict ER’s applicability in real-world systems where correlated, heterogeneous sensor networks are ubiquitous.

In summary, existing studies on correlation ER have not accounted for the correlation among heterogeneous evidence, while the constructed frameworks heavily rely on distributional assumptions. Consequently, identifying a correlation measure capable of addressing dependencies between heterogeneous evidence without requiring data to conform to specific distributions remains a critical challenge in current correlation ER research. The MIC has been recognized as a robust measure for variable relationship analysis. Its effectiveness has been demonstrated in big data environments for multi-correlation identification. MIC’s functional independence has been established, capable of detecting both linear/nonlinear correlations and complex relational patterns [29]. A MIC-based feature selection framework is introduced in [30] for IoT data processing optimization. MIC quantification was implemented to evaluate feature–class correlations and redundancy levels. Nonlinear association discovery is enabled by Liu et al. [31] through MIC-based rule mining. An enhanced MIC estimation algorithm (Back MIC) was developed by Cao et al., where algorithmic optimization is achieved through equipartition axis backtracking mechanisms [32]. A MIC-enhanced random forest algorithm was formulated in [33] to overcome computational inefficiency, feature redundancy, and expressive feature selection limitations. Parallel implementation was realized through Spark platform integration.

The current limitations are mainly in two directions: (1) Conventional ER fails to address interdependencies between correlated evidence; (2) existing ER studies that partially consider evidence correlations exhibit critical shortcomings, including heavy reliance on predefined data distribution assumptions and neglect of heterogeneous evidence interactions. This study aims to resolve these challenges by (1) developing a generalized ER framework capable of reasoning with correlated evidence and (2) introducing a data-agnostic correlation quantification mechanism that eliminates distributional assumptions while explicitly addressing heterogeneous evidence correlations. Therefore, the MICER rule framework is proposed, integrating MIC with evidential reasoning principles to enable hybrid evidence correlation analysis and inference process optimization. Three principal innovations distinguish this work from the correlation ER methodology documented in [26]:

(1) Heterogeneous evidence correlations are systematically addressed, encompassing both linear and nonlinear relationships;
(2) Evidence derivation is formulated through joint probabilistic modeling, with MIC being employed for correlation quantification;
(3) The evidence inference rule is improved from the point of view of probabilistic reasoning, the maximum mutual information coefficient evidence inference rule is proposed, and its general and recursive forms of inference procedure are given. The method is also applied to fault diagnosis.

The remainder of this paper is organized as follows. The problems with evidence-related ER rules are described in Section 2. ER rules with maximum mutual information coefficients are proposed in Section 3. A case study is carried out in Section 4 to demonstrate how the proposed ER rule is implemented and to confirm its efficacy in engineering practice. In Section 5, this paper comes to an end.

## 2. Problem Statement

### 2.1. Related Work

This section briefly explains the non-independent ER rule framework (Maximum Likelihood Evidential Reasoning, MLER Rule) in [26]. The specific reasoning process of this framework is as follows:

The first step is to obtain evidence from the data. The literature was obtained through likelihood analysis in the following steps:

**Step 1:** Create a frequency record for every input into the system:

(1) $$F_{H,i,j}=\left\{\begin{array}{l} T_{H,j,i}+T_{H,j,i+1}+T_{i,j+2s},i=1\\ T_{H,i-1,j}+T_{H,i,j}+T_{i,j+2s},i=2 \end{array}\right.,$$

where $F_{H,i,j}$ is the frequency that $x_{i}$ is in the state of j given state H, and $T_{H,j,i}$ represents the total observations of sample data corresponding to state H when system input $x_{1}$ is in the state of i and $x_{2}$ is in the state of j. s fulfills the subsequent requirement:

(2) $$s=\left\{\begin{array}{l} 0,H=H_{1}\\ 1,H=H_{2}\\ 2,H=\Theta \end{array}\right.,$$

where H is a subset of the frame of discernment (FoD) Θ.

**Step 2:** Each system input’s likelihood record is created:

(3) $$c_{H,i,j}=p((x_{i}=x_{i,j})\left|H\right.)=\frac{F_{H,i,j}}{\sum_{t=1}^{2}F_{H,i,t}},$$

where $x_{i}=x_{i,j}$ means that $x_{i}$ is in state j. $c_{H,i,j}$ are the possibilities of $x_{i}=x_{i,j}$ given state H. p(•|•) denotes the conditional probability function.

**Step 3:** Each system input’s fundamental probability record is produced:

(4) $$\beta_{H,i,j}=\frac{c_{H,i,j}}{\sum_{A\subseteq \Theta }c_{A,i,j}},$$

where $\beta_{H,i,j}$ is the basic probability that evidence $e_{i,j}$ points to state H, and A is a subset of Θ.

Based on (4), the evidence can be denoted as follows:

(5) $$e_{i,j}=\left\{(H,\beta_{H,i,j})\forall H\subseteq \Theta ,\sum_{H\subseteq \Theta }\beta_{H,i,j}=1\right\},$$

where $e_{i,j}$ refers to a piece of evidence acquired from the system input $x_{i}$ at $x_{i}=x_{i,j}$.

Then, combining the marginal likelihood function and the joint likelihood function, the calculation of the joint basic probability is given by [26].

(6) $$\beta_{H,ij,mn}=\frac{c_{H,ij,mn}}{\sum_{A\subseteq \Theta }c_{A,ij,mn}}\forall H\subseteq \Theta ,$$

where $c_{H,ij,mn}$ is the joint likelihood that both $x_{i,j}$ and $x_{m,n}$ are observed given state H. $c_{H,ij,mn}$ can be derived in the next two steps.

**Step 1:** $x_{i}$ and $x_{m}$ create a joint frequency record:

(7) $$F_{H,ij,mn}=T_{H,j,n}\forall H\subseteq \Theta ,$$

where $F_{H,ij,mn}$ is the joint frequency that $x_{i}=x_{i,j}$ and $x_{m}=x_{m,n}$ are satisfied given state H.

**Step 2:** The combined likelihood record of $x_{i}$ and $x_{m}$ is created:

(8) $$c_{H,ij,mn}=p((x_{i}=x_{i,j},x_{m}=x_{m,n})\left|H\right.)=\frac{F_{H,ij,mn}}{\sum_{u=1}^{2}\sum_{v=1}^{2}F_{H,iu,mv}},$$

For joint likelihood inference, ref. [26] gives the computation of the interdependence index on the evidence:

(9) $$\alpha_{H,ij,mn}=\left\{\begin{array}{ll} 0, & \mathrm{if}\;\beta_{H,i,j}=0\;\mathrm{or}\;\beta_{H,m,n}=0\\ \frac{\beta_{H,ij,mn}}{\beta_{H,i,j}\beta_{H,m,n}}, & \mathrm{otherwise} \end{array}\right.,$$

where $\alpha_{H,ij,mn}$ is used to measure the interdependence of $x_{i,j}$ and $x_{m,n}$. The basic probabilities of evidence $e_{i,j}$ and $e_{m,n}$ are denoted by $\beta_{H,i,j}$ and $\beta_{H,m,n}$.

After gathering the evidence’s interdependence index, a new ER rule is developed to combine the interdependent evidence as probability inference. This is how it is computed:

(10) $$\beta_{H}=\left\{\begin{array}{ll} 0, & H=\phi \\ \frac{{\tilde{m}}_{H,ij,mn}}{\sum_{A\subseteq \Theta }{\tilde{m}}_{A,ij,mn}}, & H\neq \phi \end{array}\right.,$$

(11) $$\begin{array}{ll} {\tilde{m}}_{H,ij,mn} & =[(1-r_{m,n})m_{H,i,j}+(1-r_{i,j})m_{H,m,n}]\\ & +\sum_{B\cap C=H}\gamma_{H,ij,mn}\alpha_{H,ij,mn}m_{B,i,j}m_{C,m,n}\forall H\subseteq \Theta \end{array},$$

where $\beta_{H}$ is the joint probability that $e_{i,j}$ and $e_{m,n}$ jointly support H. ${\tilde{m}}_{H,ij,mn}$ is the non-normalized combined probability mass of $e_{i,j}$ and $e_{m,n}$. $r_{i,j}$ and $r_{m,n}$ denote the reliabilities. $\gamma_{H,ij,mn}$ is a non-negative coefficient measuring the joint support from $e_{i,j}$ and $e_{m,n}$. $m_{H,i,j}$ and $m_{H,m,n}$ represent the basic probability masses that $e_{i,j}$ and $e_{m,n}$ support H, respectively. B and C are also subsets of Θ.

After the combination of two pieces of evidence $e_{i,j}$ and $e_{m,n}$, the expected utility can be calculated as follows:

(12) $$u(e(2))=\sum_{H\subseteq \Theta }\beta_{H}u(H),$$

where u(H) is the expected utility of state H. e(2) is the combination of two pieces of evidence $e_{i,j}$ and $e_{m,n}$.

While the article by Tang et al. does not take into account the diversity of evidence in the same complex system when calculating the relevance of evidence, this paper takes this into account and aims to establish a more generalized approach to considering evidence relevance in the reasoning of ER rules.

### 2.2. Evidence-Related ER Rules Problem Description

The problem description of the ER rule where evidence is not independent is as follows.

**Problem 1: (Correlation Heterogeneity):** Evidence diversity in complex systems introduces computational interference in correlation coefficient determination when inter-evidence correlations exist, resulting in biased diagnosis. This necessitates advanced correlation modeling capable of simultaneous linear/nonlinear relationship characterization.

**Problem 2: (Uncertainty Propagation):** Multi-source fusion processes are inherently affected by hybrid uncertainties in system test data. Uncertain data analysis becomes a critical research priority for probabilistic reasoning systems. While the ER framework has been widely adopted for uncertainty management, its inherent independence assumption remains problematic in practical engineering contexts. System complexity and functional interdependencies frequently violate the evidence independence requirement mandated by conventional ER implementations. Consequently, evidence correlations are systematically unaddressed in traditional ER frameworks, and the reliability of the fault diagnosis results is compromised. This work therefore focuses on reasoning process enhancement under correlated evidence conditions.

**Figure 1:**
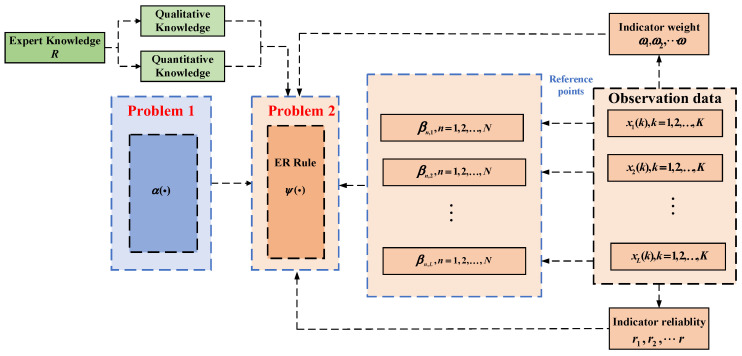
Evidence-related evidential reasoning rule structure.

The evidence-related ER rule is structured as follows (Figure 1).

The modeling of expert knowledge R includes the following. (1) For quantitative information $x_{i}$, $a_{i,j}$ denotes the jth reference value of $x_{i}$, and $a_{i,j+1}\geq a_{i,j},i=1,\ldots ,N,j=1,\ldots ,J$, where N denotes the amount of quantitative information and J denotes the number of reference values. The expert establishes a mapping relationship between the numerical quantities $x_{i,j}$ and the reference values $a_{i,j}$, i.e., $x_{i,j}\;means\;a_{i,j}$. Let $a_{i,J}$ and $a_{i,1}$ be the largest and the smallest reference values, respectively, then $x_{i}$ can be converted to the following belief distribution: $S(x_{i})=\{(a_{i,j},\beta_{i,j}),i=1,\ldots ,N,j=1,\ldots ,J\}$, where $\left\{\begin{array}{l} \beta_{i,j}=\frac{a_{i,j+1}-x_{i,j}}{a_{i,j+1}-a_{i,j}},\beta_{i,j+1}=1-\beta_{i,j},a_{i,j}\leq x_{i,j}\leq a_{i,j+1},j=1,\ldots ,J-1\\ \beta_{i,k}=0,k=1,\ldots ,J,k\neq j,j+1 \end{array}\right.$. Assuming that the reference value $a_{i,j}$ is equivalent to the evaluation level $A_{i,j}$ of the belief distribution, the quantitative information can be uniformly expressed as $S(x_{i})=\{(A_{i,j},\beta_{i,j}),i=1,\ldots ,N,j=1,\ldots ,J\}$, where $\beta_{i,j}$ denotes the belief degree that the quantitative information $x_{i}$ is evaluated as the level $A_{i,j}$. (2) For qualitative information, there is subjective judgment by experts in combination with domain knowledge to give a belief degree relative to the reference values.

### 2.3. Evidence-Related ER Framework

#### 2.3.1. Correlation Coefficient Calculation

Due to the variety of data types in actual engineering, including linear data and nonlinear data, there are a lot of irrelevant or redundant data in these data, and irrelevant or redundant data often affect the evaluation results. Therefore, it is important to choose an appropriate correlation coefficient calculation method, which must be able to handle both linear and nonlinear data. The correlation coefficient calculation process is as follows:

(13) $$\alpha =corr(x_{i},x_{j}),\;i,j=1,2,\ldots n,$$

where $x_{i}$ and $x_{j}$ are input, α represents the correlation coefficients, and corr(·) is the correlation coefficient calculation function.

#### 2.3.2. Establishment of Evidence-Related ER Rule Framework

After the appropriate correlation coefficient calculation method is selected, the improved ER rule using the correlation coefficient is

(14) $$y=newER(e_{i},e_{j},\alpha ,v),\;i,j=1,2,\ldots L,$$

where newER(·) is the new ER rule inference, y is the output, $e_{i}$ is ith evidence, and v is the set of parameters required by the model.

(15) v=O(newER(·),ε),

where O(·) denotes the optimization function; ε is the optimization parameter set.

To summarize, to construct an evidence-related evidential inference rule framework, it is necessary to solve new ER rules and optimize functions, weight, reliability, and parameter sets.

## 3. ER Rule with Maximum Information Coefficient

In this section, the ER rule is modeled and inferred by the maximum information coefficient. In Section 3.1, the greatest mutual information coefficient is used to compute the evidence correlation coefficient. The modeling of the maximum information coefficient ER rule (MICER Rule) and the optimization process are carried out in Section 3.2.

### 3.1. Correlation Analysis

Mutual information (MI) is the foundation upon which the maximum information coefficient is constructed. It is capable of mining non-functional dependencies between variables in great detail in addition to measuring both linear and nonlinear relationships between variables in vast amounts of data. Due to the diversity of data variables (including linear and nonlinear, etc.) in health status evaluation in actual engineering, the characteristics of the evidence obtained from the data are consistent with it. Therefore, this paper chooses MIC to measure the degree of association between evidence. The larger the MIC value between two variables, the stronger the correlation; otherwise, the weaker the correlation.

The maximum information coefficient is mainly calculated by mutual information and grid division methods. Mutual information can be regarded as the uncertainty reduced by one random variable because another random variable is known, and it is mainly used to measure the degree of association between linear or nonlinear variables. Let $S=\{s_{i},i=1,2,\ldots ,n\}$ and $T=\{t_{i},i=1,2,\ldots ,n\}$ be random variables, where n is the number of samples, then the mutual information is defined as

(16) $$MI(S,T)=\sum_{s\in S}\sum_{t\in T}p(s,t)\log\frac{p(s,t)}{p(s)p(t)},$$

where p(s) and p(t) are the marginal density functions; p(s,t) is the joint probability density function; MI(S,T) is the mutual information of variables s and t. The correlation between two variables is stronger the more mutual information there is between them.

Assuming that D is a finite set of ordered pairs, the definition partition Gr divides the value range of the variable S into a segments and divides the value range of T into b segments, and Gr is the grid of a×b. Mutual information MI(S,T) is calculated internally in grid division. There are many grid division methods with the same a×b, and they take the maximum value of MI(S,T) in different divisions as the value of mutual information of the division Gr. The formula for the maximum mutual information of D under the division Gr is defined as

(17) $$\overset{\wedge }{MI}(D,a,b)=\max MI(D|Gr),$$

where D|Gr means that the data D is divided using Gr. $\overset{\wedge }{MI}(D,a,b)$ denotes the maximum mutual information value of data D under a×b delimitation. MI(D|Gr) denotes the mutual information value of data D under Gr delimitation.

Even though the maximum information coefficient uses mutual information to represent the grid’s quality, it is not simply to estimate the mutual information. The maximum normalized M values obtained under different divisions form a feature matrix, and the feature matrix is defined as $Matrix{\left(D\right)}_{a,b}$, and the calculation formula is

(18) $$Matrix{\left(D\right)}_{a,b}=\frac{\overset{\wedge }{MI}(D,a,b)}{\log\min(a,b)},$$

Then, the maximum information coefficient is defined as

(19) $$\begin{array}{ll} MIC{\left(D\right)}_{a,b} & =\underset{a*b<B(n)}{\max}Matrix{\left(D\right)}_{a,b}\\ & =\frac{\overset{\wedge }{MI}(D,a,b)}{\log\min\{a,b\}} \end{array},$$

where B(n) is the upper limit value of grid division a×b. Generally, it is believed that the effect is best when $B(n)=n^{0.6}$, so this value is also used in the experiment.

In summary, the MIC calculation is divided into three steps:

**Step 1:** Given a and b, the maximum mutual information value is computed by gridding the scatter diagram made up of ST with a columns and j rows;

**Step 2:** The greatest mutual information value is normalized;

**Step 3:** The MIC value is determined by taking the highest value of mutual information at various scales.

**Remark** **1.** *Equations (13) and (16)–(19) are computed by default in this paper for ordered discrete variables.*

### 3.2. MICER Rule

The ER rule characterizes evidence through a reliable weighted belief distribution (WBDR) to supplement the belief distribution (BD) introduced in the D-S evidence theory and is established by performing orthogonal sum operations on the weighted belief distribution (WBD) and WBDR. It is a generalized Bayesian inference process or a general joint probabilistic inference method. Simultaneously, it offers a straightforward and reliable reasoning process that can handle a range of uncertainties.

Suppose the FoD is defined as $\Theta =\{H_{1},\ldots ,H_{N}\}$, where $H_{i}$ is the ith system state, i=1,…,N. The power set of Θ consists of $2^{N}$ subsets, described by

(20) $$\begin{array}{c} T(\Theta )=\{\emptyset ,\{H_{1}\},\ldots ,\{H_{N}\},\{H_{1},H_{2}\},\ldots ,\{H_{1},H_{N}\},\ldots \\ \left(H_{1},\ldots ,H_{N-1}),\Theta \right\} \end{array},$$

Suppose there are L pieces of evidence $\left\{e_{1},\ldots ,e_{L}\right\}$; the kth piece of evidence can be modeled as a basic probability distribution as follows:

(21) $$e_{k}=\{(H,\beta_{H,k}),\forall H\subseteq \Theta ,k=1,\ldots ,L\},$$

where H is a subset of Θ. $\beta_{H,k}$ is the belief assigned to state H by evidence $e_{k}$.

Assume that $w_{k}$ represents the weight of $e_{k}$ and that the following is the basic probability mass of $e_{k}$ supports H:

(22) $$m_{H,k}=w_{k}\beta_{H,k},$$

where $e_{k}$ is the kth piece of evidence.

Therefore, the underlying probability mass distribution of $e_{k}$ can be denoted as

(23) $$m_{k}=\{(H,m_{H,k})\forall H\subseteq \Theta ;(P(\Theta ),m_{P(\Theta ),k})\},$$

where $m_{P(\Theta ),k}$ is expressed as the remaining support determined by the weight of evidence, which means $m_{P(\Theta ),k}=1-w_{k}$. P(Θ) denotes the power set of Θ. There is $\sum_{H\subseteq \Theta }m_{H,k}+m_{P(\Theta ),k}=1$ because the equality constraint $\sum_{H\subseteq \Theta }\beta_{H,k}=1$ always holds. Therefore, (23) is a generalized probability distribution form.

We first provide the basic ER rule lemma, which follows the work of Yang and Xu [7].

**Lemma** **1** **(basic** **form** **of** **ER** **rule).** *For two correlated pieces of evidence $e_{1}$ and $e_{2}$, assuming their reliability is given by $r_{1}$ and $r_{2}$, respectively, their basic probability mass distribution is shown in (23). Then, the probability that $e_{1}$ and $e_{2}$ jointly support H can be calculated as follows:*

(24) $$\beta_{H,e(2)}=\left\{\begin{array}{ll} 0 & ,H=\emptyset \\ \frac{\hat{m_{H,e(2)}}}{\sum_{A\subseteq \Theta }\hat{m_{A,e(2)}}} & ,H\neq \emptyset \end{array}\right.,$$

(25) $$\begin{array}{ll} \hat{m_{H,e(2)}} & =[(1-r_{1})m_{H,1}+(1-r_{2})m_{H,2}]\\ & +\sum_{C\cap D=H}(1-MIC_{H}(e(2)))m_{C,1}m_{F,2},\forall H\subseteq \Theta \end{array},$$

*In (24), *$\beta_{H,e(2)}$* is the joint probability that *$e_{1}$* and *$e_{2}$* jointly support *H. $\hat{m_{H,e(2)}}$* denotes the unnormalized total probability mass of *H* from *$e_{1}$* and *$e_{2}$*. The symbol *A* is as a subset of *Θ*. In (25), *$r_{1}$ *and *$r_{2}$ *denote the reliabilities of *$e_{1}$* and *$e_{2}$*. The first term *$(1-r_{1})m_{H,1}+(1-r_{2})m_{H,2}$* is called the bounded sum of the individual supports of *H* by *$e_{1}$* and *$e_{2}$*, respectively. The basic probability mass that *$e_{1}$* and *$e_{2}$* support *H* is indicated by *$m_{H,1}$* and *$m_{H,2}$*, respectively. *$MIC_{H}(e(2))$* denotes the interdependence of *$e_{1}$* and *$e_{2}$*. In addition, *$\sum_{C\cap D=\theta }(1-MIC_{H}(e(2)))m_{C,1}m_{F,2}$* is called the orthogonal sum of the common support of *$e_{1}$* and *$e_{2}$*, measuring the degree of all intersected supports on proposition *H*. Symbols *C* and *F* are also arbitrary subsets of *Θ*.*

We provide the following lemma for the recursive ER rule to generalize:

**Lemma** **2** **(recursive** **form** **of** **the** **ER** **rule).** *For L pieces of relevant evidence $\left\{e_{1},\ldots ,e_{L}\right\}$, assuming their reliability is given by $\left\{r_{1},\ldots ,r_{L}\right\}$, their basic probability mass distribution is represented like in (23). Then, by iteratively using the following equation, the likelihood that L independent pieces of evidence support H overall can be produced: *

(26) $$\beta_{H,e(j)}=\left\{\begin{array}{ll} 0 & ,H=\emptyset \\ \frac{\hat{m_{H,e(j)}}}{\sum_{B\subseteq \Theta }\hat{m_{B,e(j)}}} & ,H\neq \emptyset \end{array}\right.,$$

(27) $$m_{H,e(j)}=\left\{\begin{array}{ll} 0 & ,H=\emptyset \\ \frac{\hat{m_{H,e(j)}}}{\sum_{B\subseteq \Theta }\hat{m_{B,e(j)}}+\hat{m_{P(\Theta ),e(j)}}} & ,H\neq \emptyset \end{array}\right.,$$

(28) $$\begin{array}{ll} \hat{m_{H,e(j)}} & =[(1-r_{j})m_{H,j}+m_{P(\Theta ),e(j-1)}m_{H,j}]\\ & +\sum_{C\cap D=\theta }(1-MIC_{H}(e_{j-1},e_{j}))m_{C,e_{j-1}}m_{F,e_{j}},\forall H\subseteq \Theta \end{array},$$

(29) $$\hat{m_{P(\Theta ),e(j)}}=(1-r_{j})m_{P(\Theta ),e(j-1)},$$

*where $\beta_{H,e(j)}$ represents the joint probability that the first j pieces of evidence $e_{j}$ supports H, j=2,…,L. $m_{H,e(j)}$ is the joint probability mass assignment of the first j pieces of evidence to H. $\hat{m_{H,e(j)}}$ and $\hat{m_{P(\Theta ),e(j)}}$ represent the unnormalized combined probability mass of H and Θ in the previous j pieces of evidence. There are $m_{H,e(1)}=m_{H,1}$ and $m_{P(\Theta ),e(1)}=m_{P(\Theta ),1}=1-r_{1}$, respectively. B, C, and D are also subsets of Θ.*

*The aforementioned analysis indicates that new synthetic evidence $e_{K}$ can be produced by combining the K pieces of evidence. Its probability distribution looks like this:*

(30) $$e_{K}=\{(H,\beta_{H,e_{K}})\forall H\subseteq \Theta \},$$

*where $\beta_{H,e(K)}$ is the joint probability that K pieces of evidence support H. The expected utility of e(K) is computed as follows, assuming that the utility of state H is u(e(K)):*

(31) $$u(e(K))=\sum_{H\subseteq \Theta }\beta_{H,e(K)}u(H)$$

**Remark** **2.** *Parameter definitions and methodological considerations.*

The parameters addressed in this subsection are weight and reliability of evidence and reference level. The parameters of evidence weight and reliability serve distinct roles: weights represent the subjective importance of evidence (e.g., decision-maker preferences for specific indicators), while reliability quantifies its objective credibility (e.g., sensor measurement precision or expert judgment consistency). There are many methods in setting the weight of evidence, including the subjective assignment method (e.g., expert experience method, preference rule mapping), the objective calculation method (e.g., information entropy method, data-driven method), the combination weighting method (subjective-objective combination of weights), and the dynamic adjustment mechanism (e.g., conflict feedback adjustment, utility-sensitive weights). Methods of setting reliability are based on information consistency calculations (e.g., fluctuation analysis, perturbation factor method models), modeling of statistical properties (e.g., probability density function method, interval reliability), and expert calibration methods (e.g., reliability scoring, cross-validation methods). Different weights and reliability setting methods apply to different scenarios, so it is necessary to choose suitable setting methods according to specific application scenarios when setting weights and reliability. In this paper, reference levels are set by experts in conjunction with domain knowledge.

**Remark** **3.** *Algorithmic robustness and parameterization strategy.*

The orthogonal sum operator in evidential reasoning (ER) inherently embeds a residual belief assignment mechanism that redistributes residual uncertainty to the identification framework, providing strong resistance to interference perturbations. This intrinsic robustness, empirically validated in prior studies [33,34], buffers against weight variations—experiments demonstrate ≤ 5% confidence deviation under ±20% weight perturbations. Consequently, the current methodology intentionally excludes weight optimization. Similarly, reliability calibration (reflecting objective evidence credibility) falls beyond conventional ER optimization scopes. Reference grades, derived from domain expert knowledge in this study, are retained without optimization to preserve interpretability and operational transparency. These deliberate design choices align with industrial diagnostic requirements where explainability and stability outweigh marginal accuracy gains from parametric tuning.

## 4. Case Study

### 4.1. Case of Flange Ring Loosening Fault Diagnosis

This section verifies the effectiveness of the proposed ER rule framework through a case study of flange ring loosening fault diagnosis. In optical fiber communication, signals are transmitted through optical fibers, and optical fiber connectors are responsible for connecting the transmission between optical fibers. If the flange ring in the fiber optic connector is loose, it may cause the following problems:

(1) **Signal loss:** Loose fiber optic flanges will lead to unstable connections between fibers, resulting in the failure of normal signal transmission and signal interruption.
(2) **Decrease in signal quality:** Loose fiber optic flanges may result in incomplete connection between fibers, resulting in a decrease in signal quality. This can manifest as issues such as reduced signal strength, increased noise, or data loss.
(3) **Increased refraction loss:** Loose fiber optic flanges will increase the refraction loss of light at the joint, reducing signal transmission efficiency.

**Figure 2:**
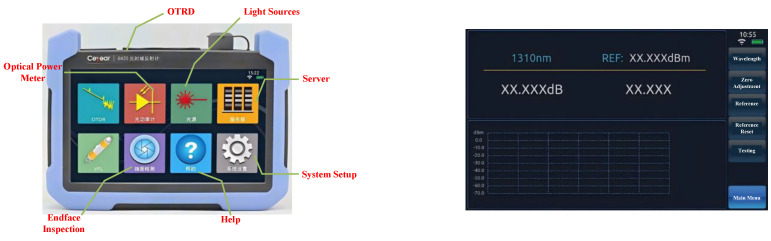
The OTDR interface.

**Figure 3:**
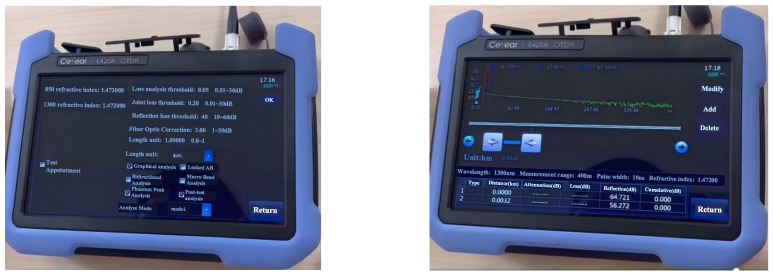
The OTDR parameter setting interface.

**Figure 4:**
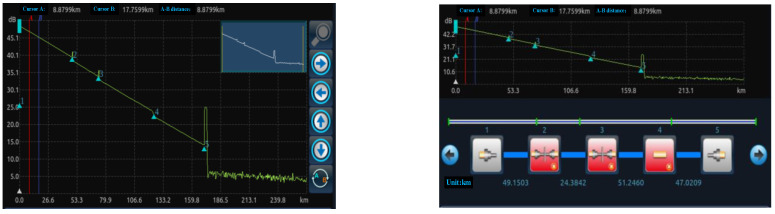
The OTDR analysis interface.

**Figure 5:**
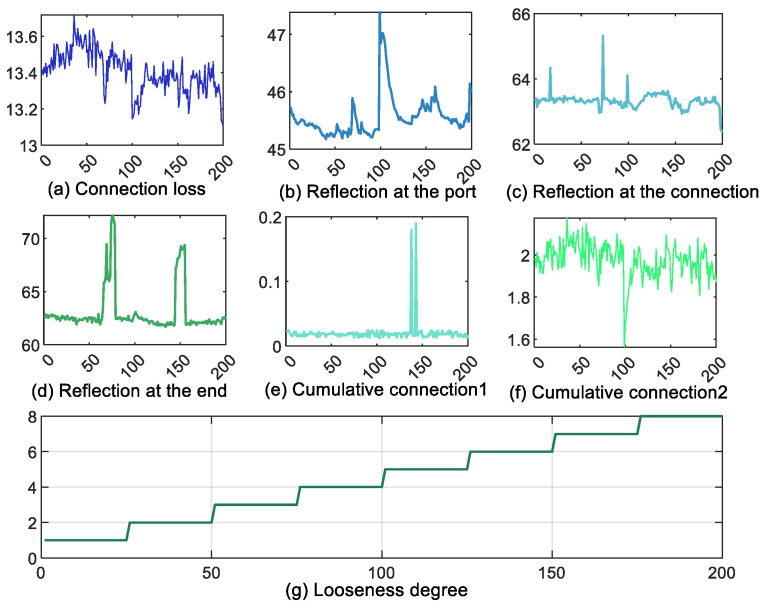
The experimental data.

In this section, a simulation experiment is carried out using a 6420A optical time domain reflectometer (OTDR). The optical sensor is used to convert the optical signals emitted by the laser into electrical signals to obtain 200 sets of data measuring the transmission loss, splice loss, and other physical characteristics of the optical fiber. The OTDR interface is shown in Figure 2, the OTDR parameter setting interface is shown in Figure 3, and the OTDR analysis interface is shown in Figure 4. The working principle of OTDR is that the light pulses emitted by the laser light source first propagate along the optical fiber, and part of the energy is returned to the OTDR port due to Rayleigh scattering and Fresnel reflection. The slope of Rayleigh scattering characterizes the fiber attenuation coefficient, while the Fresnel peaks reflect identify connectors, breakpoints, and other events. The returned scattered and reflected light is then directed to a photodetector through the directional coupler, which converts the optical signal to an electrical signal. Finally, after setting the parameters, the return curve, connection loss, reflection, and cumulative loss are displayed on the LCD screen through analysis and calculation. Therefore, when the flange ring of the optical fiber interface is loose, the propagation of light pulses will be affected, and the connection loss along the optical fiber and the reflections at various locations will also change. At the same time, combined with domain knowledge, the attributes affected by the loosening of the flange ring are found to include connection loss, reflection at the port, reflection at the connection, reflection at the end, and cumulative connection (including two types, as two attributes are considered) and looseness degree; these six attributes are utilized in this section to assess the faults of the fiber flange ring looseness, with the degree of looseness being the final diagnosis. The experimental data are shown in Figure 5.

**Figure 6:**
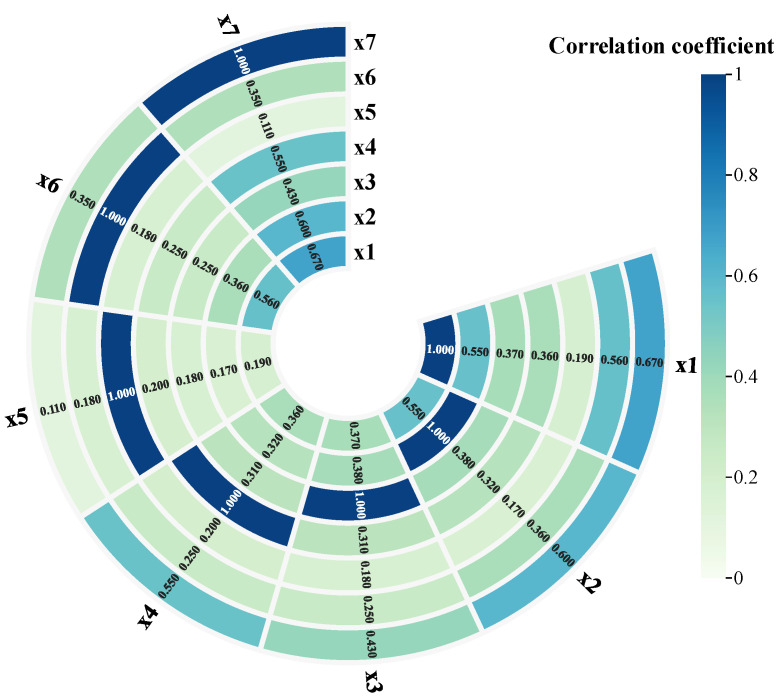
MIC value plot among the evidence.

The obtained data use the maximum mutual information coefficient to calculate the correlation coefficient of each evidence, as shown in Figure 6. The meanings of the letters in the figure are shown in Table 1.

We converted the experimental data into the corresponding belief distribution and selected some data as shown in Table 2.

**Figure 7:**
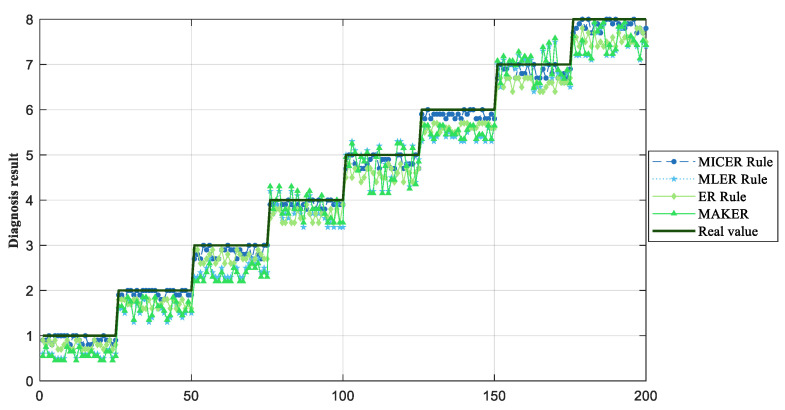
Comparison of diagnosis results of flange ring loosening.

**Figure 8:**
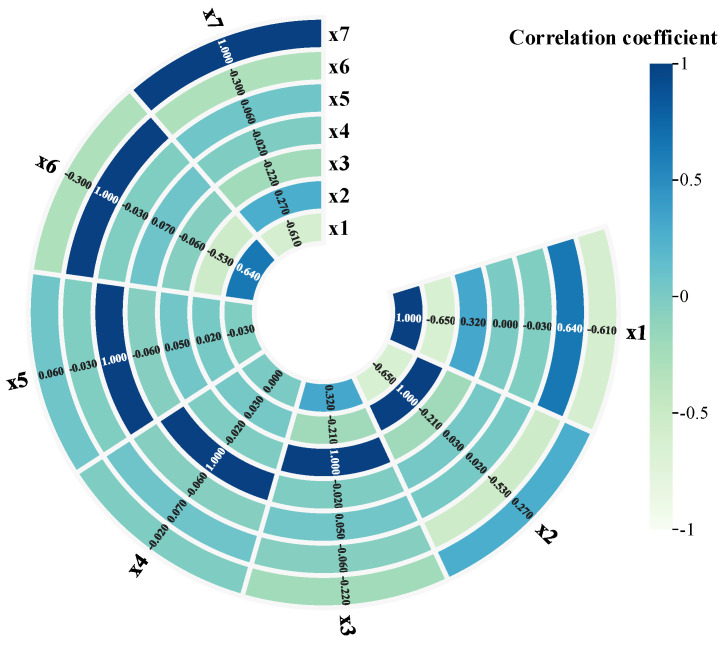
The correlation coefficient calculated by the MLER rule in flange loosening diagnosis.

Eventually, the MICER Rule model in Section 3.2 is built and reasoned through, while the MLER Rule method in Section 2.1, maximum likelihood evidential reasoning (MAKER) [35], and the traditional ER rule method are used as a comparative experiment, and a comparison of the final diagnosis results of the three methods is shown in Figure 7. The correlation coefficient calculated by the MLER rule is shown in Figure 8.

As shown in Figure 7, the proposed method achieves superior fault diagnosis accuracy compared to the MLER rule framework and MAKER. This performance advantage is attributed to the hybrid linear/nonlinear data characteristics being effectively addressed in correlation coefficient computation, a critical limitation of the MLER and MAKER approaches. Concurrently, traditional ER rule implementation methods also produce lower troubleshooting accuracies due to the failure to consider the interdependencies between evidence. The proposed methodology’s validity has been rigorously validated through comprehensive case studies encompassing both modeling and reasoning processes.

### 4.2. Case of Flywheel System Fault Diagnosis

**Figure 9:**
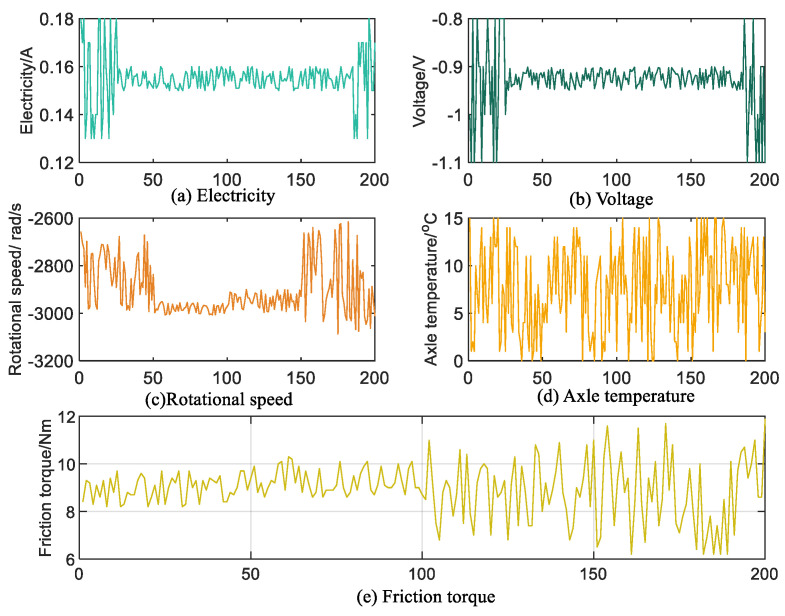
Experimental data of flywheel system.

To verify the generalization ability of the model, the flywheel system friction torque fault is selected for the fault diagnosis case study in this paper. In the acquisition of indicators for flywheel system fault diagnosis, two observations of shaft temperature and friction torque can be obtained through temperature sensors and torque sensors deployed on the bearings, while three observations of current, voltage, and speed can be obtained through the motor. The friction torque failure of the flywheel system will directly affect the change rule of the shaft temperature and rotational speed of the flywheel. Friction generates heat, which can rapidly raise the temperature of the bearings, while the rotational speed of the flywheel changes weakly due to closed-loop control. To overcome the impact of friction torque on the motor output torque, the control system will increase the voltage and current of the flywheel. At the same time, it causes the motor output torque to increase, and the motor output torque is proportional to the flywheel speed. Ultimately, the closed-loop control stabilizes the flywheel speed around the target value, while the rotational speed is affected by current and voltage. The health and fault data of the flywheel used in this paper are the telemetry and simulated values of the flywheel observation variables, as shown in Figure 9.

**Figure 10:**
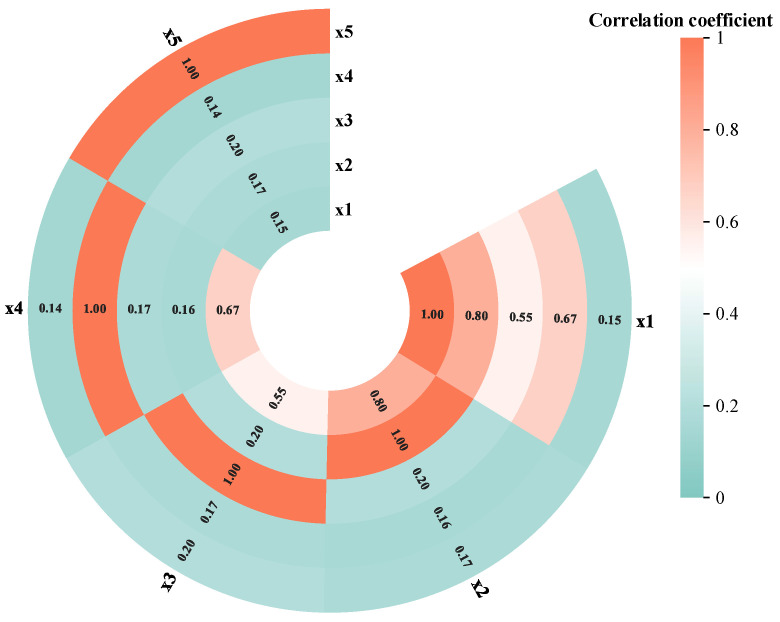
Evidence correlation coefficient of flywheel system.

The obtained data were used to calculate the correlation coefficients of each component using the maximum mutual information coefficient, as shown in Figure 10.

The model in this paper converts the experimental data into corresponding belief distributions, some of which were selected as shown in Table 3.

**Figure 11:**
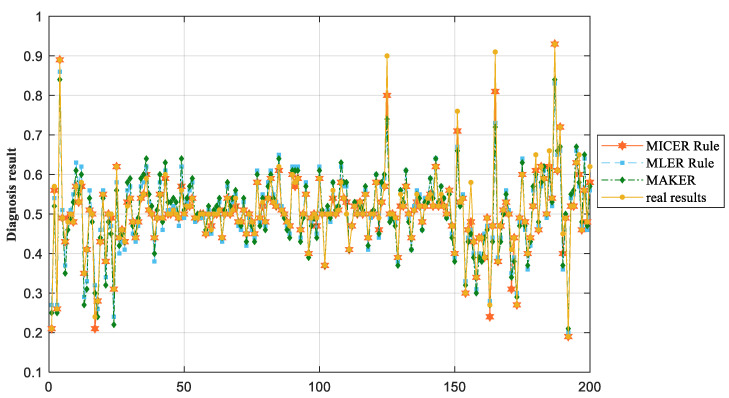
Comparison of flywheel system fault diagnosis results.

**Figure 12:**
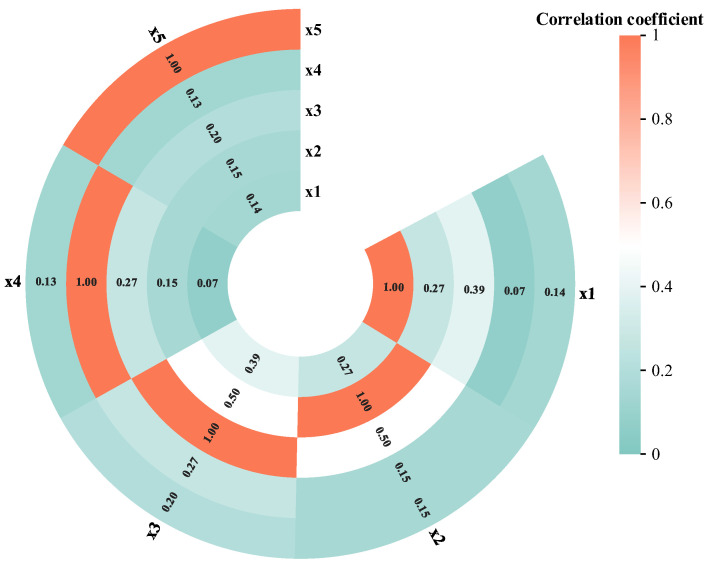
The correlation coefficient calculated by the MLER rule in flywheel system fault diagnosis.

Eventually, the MICER Rule model in Section 3.2 is built and reasoned through, while the MLER Rule method in Section 2.1 and MAKER are used as a comparison experiment, and the comparison of the final fault diagnosis results of the two methods with the real values is shown in Figure 11. The correlation coefficients calculated by the MLER Rule are shown in Figure 12.

As demonstrated in Figure 11, enhanced diagnostic accuracy is achieved by the proposed methodology, exhibiting significantly closer alignment with truth values compared to the MLER Rule framework and MAKER. The method proposed in this section is capable of both health evaluation and fault diagnosis, further demonstrating the generalization of the model.

## 5. Conclusions

This study proposes the MICER framework to address two critical limitations of conventional evidential reasoning (ER) in fault diagnosis: the independent assumption of evidence and the inability to quantify nonlinear evidence correlations. The key methodological contributions are systematically summarized as follows.

**1. Unified Correlation Analysis for Heterogeneous Evidence**

The framework introduces a novel approach to simultaneously process linear and nonlinear interdependencies among multi-source sensor data. By integrating maximum mutual information coefficient (MIC) theory, it overcomes the constraints of currently available ER methods that consider correlation, effectively capturing complex interaction patterns (e.g., inverse temperature–humidity relationships in fault diagnosis where environmental effects need to be taken into account) that traditional linear metrics fail to detect.

**2. Joint Probability Modeling with MIC Quantification**

A nonparametric joint probability modeling mechanism is established through MIC’s adaptive grid partitioning, enabling correlation quantification without prior assumptions on evidence distributions. This allows robust fusion of heterogeneous sensor data (vibration, thermal, chemical) under uncertainty, as validated in flywheel system diagnostics.

**3. Generalized ER Rule Reformulation**

The conventional Dempster combination rule is extended through recursive computation architectures that embed MIC-derived correlation weights. This reformulation resolves conflicts arising from unmodeled evidence dependencies while maintaining compatibility with existing ER implementations. The recursive form specifically addresses multi-stage fusion scenarios in static systems, avoiding combinatorial complexity growth.

Two critical research frontiers emerge from this work: first, the development of causal correlation discrimination frameworks to address spurious sensor data associations, particularly crucial for systems with time-varying operating conditions; second, the integration of online learning mechanisms to enable MICER’s autonomous adaptation to emerging fault patterns in evolving industrial systems. Future investigations should prioritize establishing standardized protocols for correlation hierarchy analysis and validation benchmarks for next-generation evidence fusion systems.

## Figures and Tables

**Table 1:** The correlation coefficient of each piece of evidence.

Letter	Meaning
x1	Connection loss
x2	Reflection at the port
x3	Reflection at the connection
x4	Reflection at the end
x5	Cumulative connection1
x6	Cumulative connection2
x7	Looseness degree

**Table 2:** Transformation of data from partial flange ring loosening into belief distributions.

	x1			x2			x3			x4		
*β*	0.00	0.98	0.02	0.49	0.51	0.00	0.34	0.66	0.00	0.83	0.17	0.00
	0.07	0.93	0.00	0.57	0.43	0.00	0.30	0.70	0.00	0.78	0.22	0.00
	0.00	0.94	0.06	0.63	0.37	0.00	0.48	0.52	0.00	0.83	0.17	0.00
	0.04	0.96	0.00	0.61	0.39	0.00	0.34	0.66	0.00	0.79	0.21	0.00
	0.00	0.87	0.13	0.68	0.32	0.00	0.40	0.60	0.00	0.84	0.16	0.00
	0.11	0.89	0.00	0.68	0.32	0.00	0.37	0.63	0.00	0.80	0.20	0.00
	0.00	0.93	0.07	0.74	0.26	0.00	0.34	0.66	0.00	0.80	0.20	0.00
	0.00	0.93	0.07	0.74	0.26	0.00	0.32	0.68	0.00	0.81	0.19	0.00
	0.00	0.90	0.10	0.77	0.23	0.00	0.31	0.69	0.00	0.78	0.22	0.00
	0.06	0.94	0.00	0.74	0.26	0.00	0.30	0.70	0.00	0.79	0.21	0.00
	0.00	0.77	0.23	0.73	0.27	0.00	0.34	0.66	0.00	0.81	0.19	0.00
	**x5**			**x6**						**x7**		
*β*	0.89	0.11	0.00	0.00		0.62		0.38		1.00	0.00	0.00
	0.87	0.13	0.00	0.00		0.76		0.24		1.00	0.00	0.00
	0.87	0.13	0.00	0.00		0.54		0.46		1.00	0.00	0.00
	0.90	0.10	0.00	0.00		0.69		0.31		1.00	0.00	0.00
	0.93	0.07	0.00	0.00		0.60		0.40		1.00	0.00	0.00
	0.94	0.06	0.00	0.00		0.85		0.15		1.00	0.00	0.00
	0.97	0.03	0.00	0.00		0.67		0.33		1.00	0.00	0.00
	0.93	0.07	0.00	0.00		0.75		0.25		1.00	0.00	0.00
	0.89	0.11	0.00	0.00		0.85		0.15		1.00	0.00	0.00
	0.93	0.07	0.00	0.00		0.86		0.14		1.00	0.00	0.00
	0.89	0.11	0.00	0.00		0.58		0.42		1.00	0.00	0.00

**Table 3:** Transformation of partial data from flywheel systems into belief distributions.

	Electricity x1			Voltage x2			Rotational Speed x3		
*β*	0.00	0.00	1.00	0.45	0.55	0.00	0.00	0.18	0.83
	0.00	0.34	0.66	1.00	0.00	0.00	0.00	0.37	0.63
	0.00	0.00	1.00	0.00	0.00	1.00	0.00	0.47	0.53
	1.00	0.00	0.00	1.00	0.00	0.00	0.15	0.85	0.00
	0.52	0.48	0.00	0.45	0.55	0.00	0.00	0.34	0.66
	0.00	0.34	0.66	0.00	0.00	1.00	0.55	0.45	0.00
	0.00	0.34	0.66	0.00	0.85	0.15	0.52	0.48	0.00
	1.00	0.00	0.00	0.00	0.85	0.15	0.00	0.57	0.43
	0.52	0.48	0.00	1.00	0.00	0.00	0.00	0.55	0.45
	1.00	0.00	0.00	0.00	0.85	0.15	0.06	0.94	0.00
	**Axle temperature** **x4**						**Friction torque** **x5**		
*β*	0.00		0.00		1.00		0.15	0.85	0.00
	0.86		0.14		0.00		0.00	0.84	0.16
	0.71		0.29		0.00		0.00	0.87	0.13
	0.86		0.14		0.00		0.19	0.81	0.00
	0.00		0.63		0.38		0.00	0.90	0.10
	0.00		1.00		0.00		0.08	0.92	0.00
	0.29		0.71		0.00		0.00	0.84	0.16
	0.00		0.50		0.50		0.23	0.77	0.00
	0.00		0.13		0.88		0.00	0.81	0.19
	0.00		0.00		1.00		0.15	0.85	0.00

## Data Availability

Data are contained within the article.

## Abbreviations

The following abbreviations are used in this manuscript:

ER	Evidential Reasoning
MIC	Maximum Mutual Information Coefficient
MICER Rule	ER Rule Framework Incorporating Maximum Mutual Information Coefficient
D-S	Dempster–Shafer
ERA	ER Algorithm
PSA_ER	Power Set Domains ER
ICS	Industrial Control System
ER-IRV	Evidential Reasoning Approach with Interval Reference Values
RTDC	Relative Total Dependence Coefficients
MLER Rule	Maximum Likelihood ER Rule
WBDR	Reliable Weighted Belief Distribution
BD	Belief Distribution
OTDR	Optical Time Domain Reflectometer
